# Generalized Tetanus Associated With Malaria and Probable Meningitis in a 4‐Year‐Old Patient: A Case Report in Limbe Regional Hospital, Cameroon

**DOI:** 10.1002/ccr3.71043

**Published:** 2025-09-28

**Authors:** Christian Damien Tchuisseu Ngapjang, Christian Fotso, Ronald Gobina, Philippe Albert Lingo, Quincy‐Jones Shumbang Tchumukong, Bertold Brecht Kouam, Marc Effila, Emelinda Berinyuy Nyuydzefon, Verla Vincent Siysi

**Affiliations:** ^1^ Department of Internal Medicine and Pediatrics, Faculty of Health Sciences University of Buea Buea Cameroon; ^2^ Intensive Care Unit Limbe Regional Hospital, South‐West Region Limbe Cameroon; ^3^ Department of Anesthesiology and Intensive Care Medicine Buea Regional Hospital Buea Cameroon

**Keywords:** children, febrile convulsion, generalized tetanus, opisthotonos

## Abstract

Tetanus is a life‐threatening disease preventable by immunization through vaccines. In resource‐limited settings, its association with other acute infections should be managed cautiously. A 4‐year‐old unvaccinated male was referred from a nearby hospital for better management of severe malaria and probable enteric fever, after presenting there with febrile seizures, neck stiffness, and vomiting. Shortly after admission, he developed trismus, recurrent generalized paroxysmal muscle spasms, and opisthotonos. Given the clinical features and lack of antitetanic immunization, a diagnosis of tetanus and cerebral malaria was established, with probable bacterial meningitis as a relevant differential. Owing to the caregiver's financial constraints, no specific investigations were carried out. The patient was admitted to the intensive care unit (ICU) and empirically treated for all three likely diagnoses, with anti‐tetanus toxoid, antibiotics, and antimalarial. Despite limited supportive care, the patient's condition considerably improved within the first 48 h in the ICU, and he spent 5 additional days in the hospital before being discharged.

**Summary**

- This case report highlights the practical challenges and management strategies for handling life‐threatening complex infection cases in resource‐limited settings.
- The minimum available resources of the patient and the health care facility have to be used wisely, prioritizing intervention, and ensuring efficient care delivery.

## 1 Introduction

Tetanus is a life‐threatening infectious disease with a serious outcome if not detected and treated at an early stage. It is caused by *Clostridium tetani* , an anaerobic bacterium that produces tetanospasmin, a neurotropic toxin [1]. The clinical features include progressive generalized rigidity and skeletal muscle spasms [2]. Diagnosis is primarily clinical, and management must be initiated promptly without awaiting paraclinical investigations. The differentials may themselves constitute immediate life‐threatening conditions. In resource‐limited settings, therapeutic interventions for tetanus disease must be adequate and adapted [3]. Here we report a case of generalized tetanus in a 4‐year‐old male with clinical presentation and evidence of severe malaria and meningitis at the Regional Hospital in Limbe, Cameroon.

## 2 Case History/Examination

A 4‐year‐old male who presented with generalized body weakness, fever, and vomiting 2 days prior to consultation at a district hospital. He was admitted and started on empiric treatment for severe malaria and salmonellosis with intravenous Artesunate, Ceftriaxone, and Gentamicin. A few hours after admission, he had multiple episodes of tonic seizures with trismus, for which he received a benzodiazepine and a barbiturate; then he was referred to our service at the Limbe Regional Hospital for better management.

**FIGURE 1:**
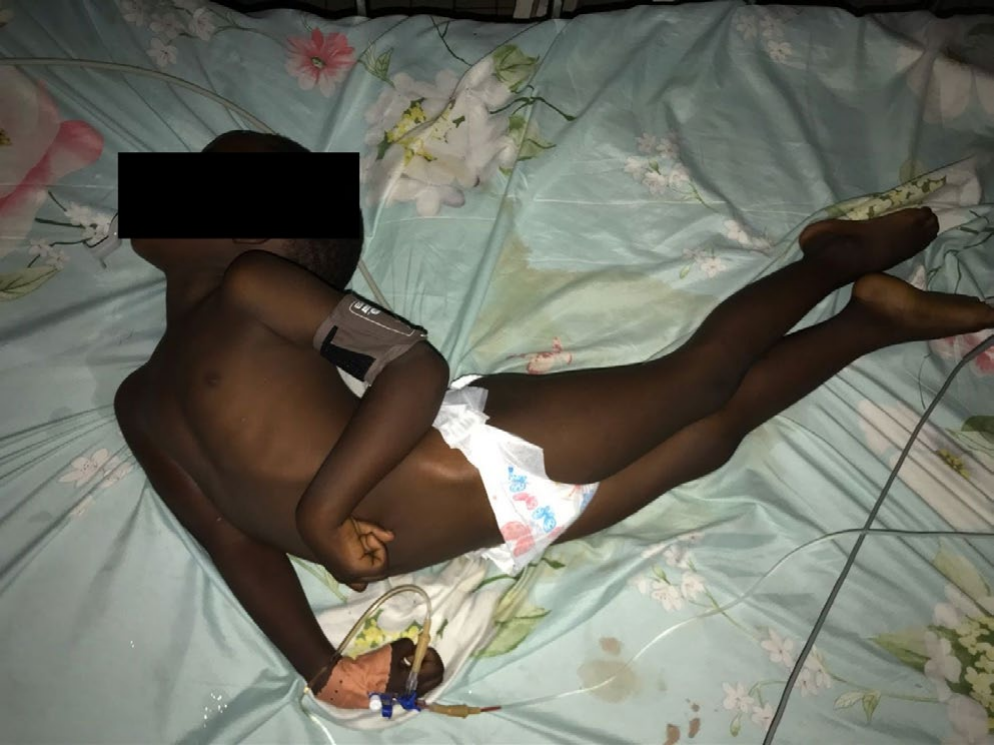
Opisthotonos position in a 4‐year‐old male with generalized tetanus on arrival at the Emergency unit—Limbe Regional Hospital, Cameroon.

On arrival, the patient exhibited high‐grade fever, pulse of 140 bpm, respiratory rate of 35 cpm, and an oxygen saturation of 96%. His conjunctivae were pale, oropharynx difficult to assess because of the jaw rigidity (trismus). The lungs and abdomen examination was unremarkable. The patient had multiple episodes of generalized contractures with opisthotonos (Figure 1). There were no wounds and no recent scars found on a meticulous skin examination. The prognostic Dakar score, as shown in Table 1, was used as a tool to assess the severity and prognosis of the patient. The patient had a score of 4/6, interpreted as a severe tetanus (Table 2). His recent history revealed no recorded recent injury, wound, or animal/insect bites. For an unknown reason, the child had not received any tetanus vaccination as recommended by the Expanded Program on Immunization (EPI) in Cameroon.

**TABLE 1:** Prognostic scoring system in tetanus: Dakar score. To be assessed 48 h after symptom onset.

Prognostic factor	Dakar score	
	Score 1	Score 0
Period of onset	< 7 days	≥ 7 days or unknown
Incubation period	< 2 days	≥ 2 days
Entry site	Umbilicus, burn, uterine, open fracture, surgical wound, intramuscular injection	All others plus unknown
Spasms	Present	Absent
Fever	> 38.4°C	< 38.4°C
Tachycardia	Adult > 120 beats/min Neonate > 150 beats/min	Adult < 120 beats/min Neonate < 150 beats/min
Total score		

**TABLE 2:** Interpretation of Dakar score: Tetanus severity.

Score	Interpretation
0–2	Mild tetanus
3	Moderate tetanus
≥ 4	Severe tetanus

Laboratory investigations showed a positive malaria parasite test with a parasite density of 11,200 trophozoites/mm^3^. Other investigations done are shown in Table 3. Cerebrospinal fluid (CSF) analysis was requested alongside blood culture, serum electrolytes, and procalcitonin but could not be done for financial reasons.

**TABLE 3:** Laboratory results on admission: Complete blood count results and parasitemia levels.

Laboratory investigations		Results		Normal values
		Eve of admission	Admission day	
Complete blood count	White blood cells	5.5	8.1	5.0–12.0 × 10^9^/L
	Lymphocytes	30.2	26.8	40.0%–70.0%
	Granulocytes	3.4	5.1	2.0–7.8 × 10^9^/L
	Hemoglobin	8.2	7.8	11.0–16.0 g/dL
	MCV	87.2	79.5	73.0–87.0 fL
	Platelets	6	70	150–400 × 10^9^/L
Parasitemia levels		11,200	—	0 trophozoites/mm^3^

## 3 Differential Diagnoses and Management

The initial differential diagnoses were generalized tetanus, cerebral malaria, and meningitis.

Treatment for malaria continued with intravenous Artesunate at 2.4 mg/kg/dose given at 12 h intervals for 7 days. In the absence of CSF analysis ruling out meningitis and in the context of severe sepsis with seizures, empirical treatment with intravenous Ceftazidime at the meningeal dose was initiated. Amikacin at 20 mg/kg/24h for 3 days was also added as an empirical protocol requirement to use bi‐therapy for severe sepsis in our setting. Anti‐tetanus immunoglobulin serum was given intravenously at 10,500 IU, and Metronidazole 10 mg/kg/6h for 10 days served to ensure *Clostridium tetani* clearance. He received an intravenous bolus of Diazepam (5 mg), after which a maintenance dose of 0.15 mg/kg/h was given via electric syringe pump as a treatment for repetitive paroxysmal tetanic crisis. The level of sedation on Diazepam was evaluated with the Ramsay Score (Table 3), and the target score was 4/6 for the first 24 h. Paracetamol was also given for temperatures > 38.5°C to fight fever and limit the risk of febrile seizure. A urinary catheter was inserted to monitor the diuresis and a nasogastric tube placed for gastric content emptying.

The patient was not intubated; oxygen was administered through nasal cannula at 4 L/min. He was placed on nil per os and a continuous infusion of Dextrose 10%. He was also transfused with 1 pint of isogroup isorhesus and cross‐matched whole fresh blood. To prevent photophobia and phonophobia, he was placed in a room with minimal light and noise.

The Ramsay Score, blood pressure, pulse, respiratory rate, temperature, oxygen level saturation, glycemia as well as the urine output was monitored.

## 4 Outcome/Follow‐Up

After 48 h of management in the intensive care unit (ICU), we noted a significant reduction in the symptoms, encouraging the gradual weaning of Diazepam. We also noted a significant decrease in body temperature (Table 4), which was considered a clinical indication of the efficiency of the antibiotics and antimalarial that were being given.

**TABLE 4:** Evolution of temperature of the patient: A progressive decrease as shown here is a clinical indication of antibiotics and antimalarial efficiency in the treatment.

	Hospitalization days						
	On paracetamol			No paracetamol			
	Day 1	Day 2	Day 3	Day 4	Day 5	Day 6	Day 7
Highest	41.0	40.7	38.5	38.5	38.5	37.8	37.2
Lowest	38.2	38.0	36.8	36.5	36.6	36.6	36.6

On day 4, the patient was stable with a better general state, no more tetanic crises, and a good neurologic state. He was transferred to the pediatric ward for further management.

The patient was discharged on day 7 and subsequently followed up as an outpatient, and no other complications were noted. The family received comprehensive counseling on malaria prevention strategies and was strongly advised to complete the recommended anti‐tetanus vaccination schedule per EPI in Cameroon.

## 5 Discussion

Tetanus is an infection caused by *Clostridium tetani* , a telluric microorganism that spreads into the body usually through a portal of entry which might be a cutaneous break‐in by superficial or deep wounds. However, in approximately 20%–30% of cases, no portal of entry is found, as the bacteria can infiltrate unnoticed and trivial wounds [4, 5, 6]. This aligns with our patient's presentation, where neither an entry wound nor a history of recent trauma was documented. Most cases of tetanus occur in developing countries, particularly regions with inadequate immunization coverage or waning immunity [7]. The incubation period ranges from 3 to 21 days (median: 14 days), but may extend to several months after wound inoculation [6, 8]. The clinical manifestations of generalized tetanus in children include paroxysmal muscle spasms, neck stiffness, trismus, opisthotonos, and fever. In Cameroon, tetanus carries significant mortality: a recent study reported a 48.5% case‐fatality rate in adults [9], while another documented a fatal outcome in a 4‐year‐old with moderate tetanus (Dakar classification) [10]. Although a Dakar score ≥ 4 (severe tetanus) correlates with elevated mortality [11], our patient survived. This good outcome is highly attributable to the prompt and aggressive management that was done. The differentials of tetanus are very broad, encompassing many etiologies of febrile convulsions such as malaria and acute bacterial meningitis [12].

In Limbe, a 2021 cross‐sectional study revealed that more than one‐quarter (27.4%) of febrile children had malaria [13]. Cerebral malaria, a severe complication, is defined as an alteration of mental status, associated with the detection of *Plasmodium falciparum* in a blood sample, after excluding hypoglycemia and other causes of encephalopathy [14]. This condition is associated with a high rate of seizures [15]. In our patient, cerebral malaria was diagnosed based on high parasitemia and profound consciousness impairment.

Acute bacterial meningitis is a severe bacterial infection and the common causal pathogens in children are *Haemophilus influenzae type B*, *Streptococcus pneumoniae* , and *Neisseria meningitidis* [16]. In Cameroon, vaccination programs have significantly reduced meningitis incidence [17, 18]. Diagnosis requires clinical assessment (fever, headache, neck stiffness, altered mental status, seizures, photophobia, nausea, and vomiting) and laboratory confirmation [19]. However, early empiric antibiotic therapy is crucial and should not be delayed by paraclinical investigations [19, 20].

The patient's presentation (fever, convulsion, neck stiffness, vomiting) and uncertain vaccination status raised strong suspicion for bacterial meningitis despite diagnostic limitations in this resource‐limited setting.

The challenge with the patient was the high financial constraint limiting the paraclinical investigations for diagnostic confirmation and biological monitoring of the case. Ideally, lumbar puncture with CSF analysis would have definitively ruled out meningitis and potentially identified causative pathogens [16, 19, 20]; blood cultures could have isolated *Clostridium tetani* . These investigations would have enabled precise diagnosis, potentially avoiding unnecessary treatments that carry risks of antimicrobial resistance or adverse drug reactions [21, 22, 23]. Given the diagnostic limitations, we implemented empirical therapy covering all three potential diagnoses and their complications. Therefore, antibiotics, antimalarial, and other important supportive medications were prioritized. Definitive diagnoses were established as tetanus and malaria, while meningitis remained presumptive based on clinical presentation.

For presumed acute bacterial meningitis, the second‐line empirical antibiotic was initiated because of a lack of improvement after 2 days of first‐line therapy at the referring health facility. Considering potential infection with multiple pathogens, including gentamicin‐resistant organisms, we escalated therapy to Ceftazidime (a third‐generation cephalosporin with pseudomonal coverage) combined with Amikacin [24, 25, 26].

Artesunate was given for severe malaria according to the national treatment guidelines [27].

While international guidelines recommend intubation with mechanical ventilation and sedation for prevention of aspiration pneumonia, optimal muscle relaxation and respiratory support in tetanus cases [6, 28], resource constraints at Limbe Regional Hospital's ICU necessitated an adapted approach. With only two ventilators serving six beds, limited blood gas monitoring capabilities, staffing shortages, and unreliable electricity (untimely power shortages), the management of intubated patients is not optimal. Therefore, we prioritized non‐invasive management. Continuous intravenous Diazepam infusion, titrated via electric syringe pump to maintain a Ramsay sedation score of 4/6 [29, 30], effectively controlled spasms while preserving adequate respiratory function as supported by the literature [31]. This approach achieved satisfactory neurologic, hemodynamic, and respiratory stability without intubation. It was similar to the approach described in a study conducted in a low‐income ICU setting where close to 75% of tetanus patients were not intubated [3]. Intubation was reserved as a rescue intervention for refractory instability, balancing the risks of suboptimal ventilator management against potential benefits.

Tetanus is a vaccine‐preventable disease included in the Expanded Program on Immunization in Cameroon. However, persistent misconceptions about vaccination contribute to low immunization rates, leaving children vulnerable to tetanus and other preventable infections like bacterial meningitis [32, 33]. This challenge is particularly evident in South‐west Cameroon, where most tetanus patients lack documentation on their immunization status [34]. The antibiotic of choice for tetanus is Metronidazole, and the administration of tetanus immunoglobulin is one of the vital aspects of the management. The modalities of supportive treatment are standard and should be assessed case by case. The speedy recovery of our patient was related to the rapid diagnosis, prompt management as well as the prioritization of the actions despite limited resources.

## 6 Conclusion

Tetanus carries significant mortality in resource‐limited settings, where outcomes depend critically on prompt diagnosis and immediate intervention. In malaria‐endemic countries, an onset of generalized tetanus can be mistaken for cerebral malaria, hence treated as such. Clinical alertness and rational use of limited resources are required to optimize the management outcomes.

## Author Contributions

All the authors contributed to the design of the manuscript, the extraction of data, and the literature review. They all approved the final version of the manuscript.

## Consent

The written informed consent was obtained from the mother of the patient for publication of this case report and the accompanying image.

## Conflicts of Interest

The authors declare no conflicts of interest.

## Data Availability

Data openly available in a public repository that issues datasets with DOIs.
